# Lung transplantation in South Africa: Indications, outcomes and disease-specific referral guidelines

**DOI:** 10.7196/SARJ.2018.v24i3.217

**Published:** 2018-09-07

**Authors:** G L Calligaro, J Brink, P Williams, A Geldenhuys, M Sussman, T Pennel

**Affiliations:** 1 Division of Pulmonology, Department of Medicine, Groote Schuur Hospital and University of Cape Town, Cape Town, South Africa; 2 Centre for Lung Infection and Immunity, University of Cape Town Lung Institute, Cape Town, South Africa; 3 Chris Barnard Division of Cardiothoracic Surgery, Department of Surgery, Groote Schuur Hospital and University of Cape Town, Cape Town, South Africa; 4 Netcare Milpark Hospital, Johannesburg, South Africa

**Keywords:** lung transplantation, end-stage lung disease, cystic fibrosis, interstitial lung disease, pulmonary hypertension, chronic obstructive lung disease

## Abstract

Lung transplantation (LT) is a robust therapy for advanced lung disease, which offers recipients extended and good-quality survival. In South Africa (SA), patients have historically had limited access to this therapy, particularly if unfunded. LT has been used as a successful therapeutic intervention for a wide variety of end-stage pulmonary parenchymal and vascular diseases, but the most common diseases that lead to LT are chronic obstructive pulmonary disease, interstitial lung disease, cystic fibrosis, alpha-1-antitrypsin deficiency and pulmonary arterial hypertension. Timing of referral for LT can be challenging and is disease specific, influenced by the rate of progression of the disease, the development of associated comorbidities, and access and response to advanced therapies. Advances in recipient and donor selection, surgical technique and postoperative management have improved early survival, but mortality remains higher than for other solid organ transplants. Rejection and infection remain major causes of early posttransplant death, while chronic rejection is the major cause of death after the first year. Survival is heavily influenced by the underlying lung disease. In this review, we summarise the indications and contraindications for LT, remind pulmonologists of the availability of this therapy in SA and offer guidelines for the timely referral of suitable candidates.

## Background

In 1963, James D. Hardy performed the first human lung transplant in Jackson, Mississippi, several years before Christiaan Barnard accomplished the corresponding feat with the human heart in Cape Town in 1967.^[1]^ Early outcomes in lung transplantation (LT) were disappointing: more than two-thirds of recipients succumbed to complications from ischaemic airway anastomotic dehiscences and early graft failure, poor healing complicated by the operative technique and the use of high-dose cortisone, the only immunosuppressive drug available at the time. However, since the introduction of the steroid-sparing drug cyclosporine, advances in donor and recipient selection and improvement in the surgical procedure to preserve vascularity (implanting each lung separately with a distal bronchial anastomosis, rather than an *en bloc* implantation with a tracheal anastomosis), LT became an accepted and successful therapy for patients with end-stage lung disease. Since the early 1990s, more than 25 000 lung transplants have been performed at centres around the world, and almost 5 000 lung transplants were performed globally in 2017 alone.^[2]^

There are several reasons for LT being the last of the solid organ transplants to really gain traction as a viable treatment.

Firstly, procurement rates from deceased donors are much lower than for other organs: lungs are procured from ~20% of cadaveric donors, whereas kidneys and livers are harvested from ~90% of donors, and hearts from ~50% of donors. This difference is probably due to the lung’s vulnerability to events arising before or after brain death of the donor, such as chest trauma, aspiration, injurious ventilation, pneumonia and neurogenic pulmonary oedema.^[3]^

**Fig. 1:**
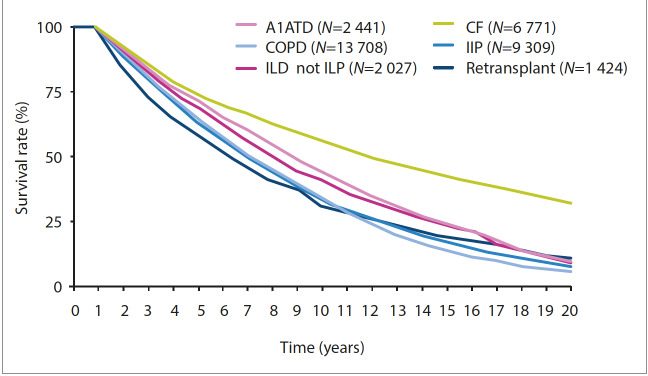
Long-term survival of adult lung transplant recipients, categorised according to diagnosis and conditional to 1-year survival. (Based on data from the International Society for Heart and Lung Transplantation registry,January 1990 - June 2016.)^[2]^ A1ATD = alpha-1-antitrypsin deficiency CF = cystic fibrosis COPD = chronic obstructive pulmonary disease IIP = idiopathic interstitial pneumonia ILD = interstitial lung disease

Secondly, outcomes have historically been much poorer: the median survival for all adult transplants is ~6 years, although this differs substantially when evaluated according to underlying diagnosis (Fig. 1).

Longer and more intensive immunosuppression is required for lung transplants than for other solid organ transplants and, despite induction immunosuppression and the use of aggressive maintenance regimens, acute allograft rejection following LT is still a significant problem.^[4]^ Furthermore, chronic lung allograft dysfunction, which usually manifests as the bronchiolitis obliterans syndrome (BOS), occurs to some degree in all patients. BOS, which is defined as a drop of >20% in forced expiratory volume in one second (FEV_1_) compared with the best achieved in the first year after transplantation, is the leading cause of mortality in transplant recipients who survive the first year, and accounts for ~30% of deaths;^[2]^ at 5 years after transplantation, the incidence of BOS is almost 50%.^[5]^

**Fig. 2:**
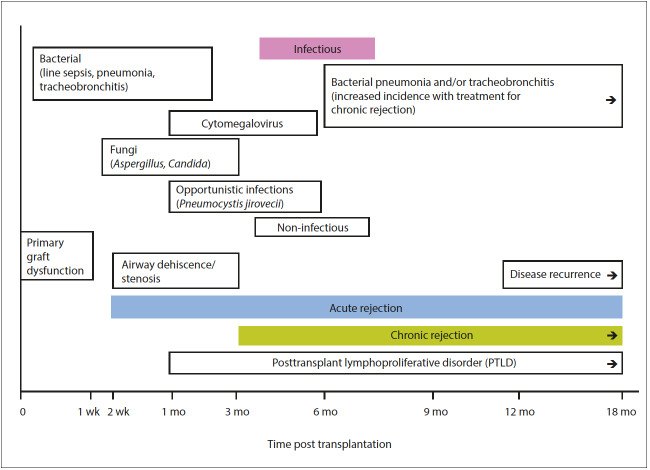
Timing of complications following lung transplantation.

Lastly, infectious complications are major contributors to morbidity and mortality in lung transplant recipients and account for more than 25% of all posttransplant deaths.^[6]^ Bacterial, viral, fungal and mycobacterial infections all occur at an increased frequency after transplantation (Fig. 2). The high levels of immunosuppression, the direct exposure of the allograft to the environment and the adverse effect of the surgical procedure on local pulmonary host defences (lymphatic disruption, altered mucociliary clearance and decreased cough) all contribute to the increased risk.

## Indications

LT should be considered in patients with chronic lung disease who are clinically deteriorating despite maximal therapy. Candidates should have a high risk of dying within the next 2 years, but no condition or combination of conditions that may result in an unacceptably high risk of mortality or morbidity, limiting the likely survival benefit from transplantation or the predicted gain in quality of life.

**Fig. 3:**
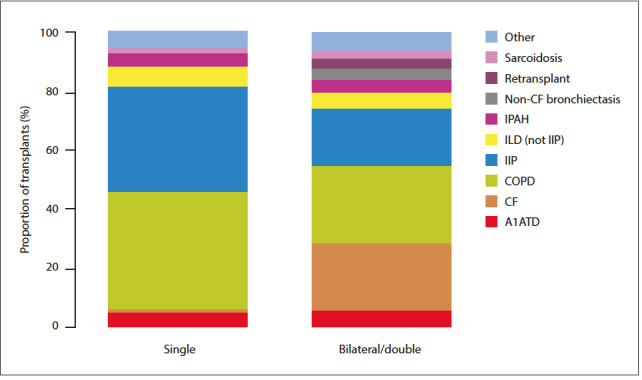
Diagnoses informing adult lung transplants, categorised according to procedure type. (Based on data from the International Society for Heart and Lung Transplantation registry,January 1990 - June 2016.)^[2]^

The most common indications for LT – accounting for ~85% of the procedures performed worldwide – are advanced chronic obstructive pulmonary disease (COPD), idiopathic pulmonary fibrosis (IPF), cystic fibrosis (CF), emphysema due to alpha1-antitrypsin deficiency and pulmonary arterial hypertension (PAH). The remaining 15% consist of a variety of diagnoses across the spectrum of end-stage lung disease, from sarcoidosis to lymphangioleiomyomatosis to pulmonary Langerhans cell histiocytosis (PLCH). The major conditions for LT globally are shown in Fig. 3, dichotomised by procedure type.

## Procedure type

**Fig. 4:**
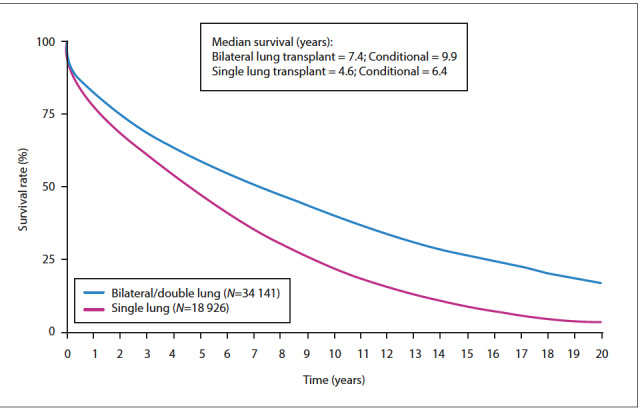
Survival rate of adult lung transplant recipients, categorised according to procedure type. (Based on data from the International Society for Heart and Lung Transplantation registry, January 1990 - June 2016.)^[2]^

A lung transplant can involve the implantation of one or both lungs. Bilateral lung transplants – essentially sequential single lung transplants with anastomoses at the mainstem bronchi – are performed preferentially in all patients, as the greater postoperative lung function provides more reserve for posttransplant complications and, in turn, is associated with better survival (Fig. 4).

 A bilateral lung transplant is always indicated in patients with suppurative lung disease (i.e. CF and bronchiectasis) and is also recommended for younger patients with COPD or alpha-1-antitrypsin deficiency. Single lung transplants have the theoretical advantage of reduced surgical morbidity (shorter duration of surgery and extracorporeal mechanical support probably not being needed, therefore avoiding the need for anticoagulation) and are often reserved for older patients with higher operative risk. Single lung transplants may be performed in any non-suppurative lung disease (where there is no fear of infection of the graft by the native lung), including IPF, sarcoidosis and PLCH, but are avoided in bullous lung disease, as preferential ventilation to the compliant native lung may cause compression of the graft and PAH. Single lung transplants also allow for optimisation of the donor pool: two separate recipients may be transplanted from a single donor and, in other cases, the contralateral lung can still be utilised if one donor lung is unsatisfactory for whatever reason.

## Contraindications

The following contraindications for LT have been compiled based on consensus and expert opinion by the International Society for Heart and Lung Transplantation:^[6]^

- active malignancy in the last 5 years for most cancers (haematologic malignancy, sarcoma, melanoma or cancer of the breast, bladder or kidney); however, a 2-year disease-free interval with a low predicted risk of recurrence may be reasonable in some cancers, such as low-grade prostate or nonmelanomatous lung cancer, although careful consultation with the patient’s oncologist is necessary in these cases.
- irreversible, significant dysfunction of other organs or body systems, including uncorrected atherosclerotic disease not amenable to revascularisation.
- some chronic infections (e.g. *Burkholderia cenocepacia* and *Mycobacterium abscessus*) if there is no viable posttransplant treatment strategy available; patients with hepatitis B or C may be suitable for LT, depending on viral load assays of peripheral blood, absence of chronic liver disease and response to antiviral eradication therapy.
- *Mycobacterium tuberculosis* infection; treated pulmonary tuberculosis is not a contraindication for LT, but may require confirmation of adequacy of therapy prior to acceptance for transplantation.
- documented non-adherence or inability to comply with complex medical therapy or office follow-up (e.g. an untreatable psychological or psychiatric condition).
- substance addiction (e.g. alcohol, tobacco or illicit drug use) that is either current or was active within the last 6 months.
- significant chest wall or spinal deformity causing severe restriction.
- body mass index (BMI) >35.0 kg/m² is an absolute contraindication; a BMI of 30.0 - 34.9 kg/m² , particularly central obesity, is a relative contraindication, which is influenced by ethnicity.
- severe or progressive malnutrition.
- severe symptomatic osteoporosis.
- complicated diabetes as indicated by established end-organ complications of microvascular disease, diffuse vascular disease and poor glycaemic control (HbA1c >8%).
- uncorrectable bleeding diathesis.
- absence of an adequate and reliable social support system.
- severely limited mobility with poor rehabilitation potential.
- although there is no absolute age limit, it is likely that the presence of multiple comorbidities in patients over 65 years of age will exclude most such patients from consideration for LT.

## Timing of the referral for lung transplantation

**Fig. 5:**
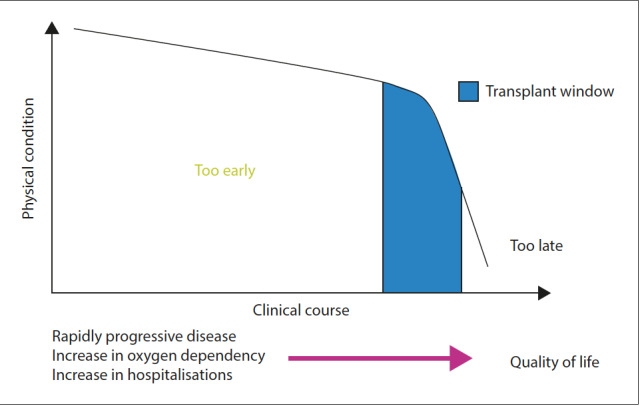
Lung transplantation referral timeline.

One of the most difficult decisions when referring a patient for LT is defining the appropriate time for transplantation (also called the transplant window – see Fig. 5).

 A patient should be sick enough to need the transplant, but not so disabled that they would not survive the procedure. Guidelines for referral for evaluation and for listing for transplantation have been established for each of the major indications for LT. These guidelines are based on expert consensus, and there are few well-designed and controlled studies that inform their use. Local expertise in management of the underlying conditions, access to advanced therapies that may delay the time to transplantation and country-specific differences in the availability of donor organs also influence these guidelines. Access to endoscopic lung volume reduction in COPD, antifibrotic medication in interstitial lung disease, potentiator/corrector treatments in CF and advanced pulmonary vasodilators in PAH is extremely limited, for both funded and unfunded patients in South Africa (SA), and so transplantation timelines may need to be substantially shortened to compensate for the lack of these therapies. Disease-specific considerations on the timing of referral for LT follow below.

### Chronic obstructive pulmonary disease

COPD is the most common indication for LT worldwide, accounting for 40% of all transplants. The variability in the natural course of COPD can make it difficult to predict when patients should be referred for LT. However, the BODE index (derived from measurements of body mass index, degree of airflow obstruction, dyspnoea score and exercise capacity) can be used to predict mortality; this can be balanced against the risk of the transplant. Suitable patients may be referred for LT when the:

- disease is progressive despite optimisation of pharmacological management, pulmonary rehabilitation and oxygen therapy.
- patient is not a candidate for endoscopic or surgical lung volume reduction surgery.
- FEV_1_ <25% of the predicted volume.
- BODE index ≥5.

### Cystic fibrosis

Predicting pretransplant survival using objective data in CF is difficult. An FEV_1_ <30% of the predicted value has been associated with a 2-year mortality of 40% in men and 55% in women.^[7]^ The number and frequency of hospitalisations and courses of home antibiotics have also been shown to be independent predictors of 2-year mortality.^[8]^ In general, a clinical decline characterised by increasing frequency of exacerbations associated with life-threatening haemoptysis, pneumothorax, increasing antibiotic resistance and recovery from a pulmonary exacerbation, a worsening nutritional status or an episode of acute respiratory failure requiring non-invasive ventilation should be a trigger for referral for LT. Other objective referral criteria in CF include:

- FEV_1_ <30% of the predicted volume, especially if a rapid downward trajectory is observed; this is seen more frequently in female patients and those with CF-related diabetes
- a 6-minute walk distance <400 m
- development of pulmonary hypertension not due to a current hypoxic pulmonary exacerbation.

### Interstitial lung disease

Owing to a rapid clinical course, patients with a confident radiological diagnosis of IPF or a histological diagnosis of non-specific interstitial pneumonia should be referred early for LT, regardless of their lung function.^[9]^ Additional considerations for referral in interstitial lung disease include:

- a decline of ≥10% in forced vital capacity and ≥15% in diffusing capacity of the lungs for carbon monoxide within the previous 6 months
- development of pulmonary hypertension
- hospitalisation because of respiratory decline, acute exacerbation or pneumothorax
- significant exercise-associated desaturation or requirement for oxygen.

### Pulmonary arterial hypertension

The development of targeted medical therapy for pulmonary hypertension (prostanoids, endothelin receptor antagonists and phosphodiesterase inhibitors) has dramatically changed decision-making around the timing of transplantation in patients with PAH and can offset the need for transplantation by many years, even indefinitely. Regrettably, few of these agents are widely available in SA, and access even for funded patients is problematic. The United States Registry to Evaluate Early and Long-term PAH Disease Management (REVEAL) found patients with high pulmonary vascular resistances, poor functional class and decreased 6-minute walk distance to be at high risk of mortality.^[10]^ Additional considerations for timing of referral for LT include the following:

- World Health Organization Functional Class III or IV during escalating vasodilator therapy or if combination vasodilator therapy cannot be accessed.
- all patients on parenteral targeted therapy (prostanoids), regardless of exercise tolerance or functional class.
- right heart catheter measurements of mean right atrial pressure >15 mmHg, a cardiac index of <2 L/minute/m² and mean pulmonary artery pressure >50 mmHg.
- known or suspected pulmonary veno-occlusive disease or pulmonary capillary haemangiomatosis.

## Lung transplantation in South Africa

The first human lung transplant in SA – a right single lung transplant – was performed at Groote Schuur Hospital in 1993. Although this was preceded by combined heart-lung transplants in the previous decade, neither therapy was enthusiastically adopted at the time, in part owing to changes in national policy around solid organ transplantation at the turn of the millennium. In 2017, LT was re-established at Groote Schuur Hospital (GSH) – the only hospital in the state sector offering thoracic transplantation – for selected patients with respiratory failure and it is, to our knowledge, the only academic centre in Africa offering this therapy to uninsured patients. GSH now has an established programme and has performed several successful bilateral lung transplants to date. In the private sector, active lung transplant programmes exist in Johannesburg and Durban, with the team at the Milpark Hospital having performed 110 transplants since the programme’s initiation in 2000. LT remains an underutilised and under-resourced therapy in SA.

## Conclusion

LT is an established therapy for advanced lung diseases for which no other therapy is applicable and can provide extended survival and excellent quality of life. It is available in both the private and state sectors in SA, albeit with regional predominances. Pulmonologists practising in our country should have an increased awareness of the availability of this therapy and of the triggers for timely referral of suitable patients.

## References

[1] Hardy JD, Webb WR, Dalton ML Jr., Walker GR Jr (1963). Lung homotransplantation in man.. JAMA.

[2] Lund LH, Khush KK, Cherikh WS (2017). The Registry of the International Society for Heart and Lung Transplantation: Thirty-fourth Adult Heart Transplantation Report-2017; Focus theme: Allograft ischemic time.. J Heart Lung Transplant.

[3] Miñambres E, Pérez-Villares JM, Chico-Fernández M (2015). Lung donor treatment protocol in brain dead-donors: A multicenter study.. J Heart Lung Transplant.

[4] Benzimra M, Calligaro GL, Glanville AR (2017). Acute rejection.. J Thorac Dis.

[5] Boehler A, Estenne M (2003). Post-transplant bronchiolitis obliterans.. Eur Respir J.

[6] Weill D, Benden C, Corris PA (2015). A consensus document for the selection of lung transplant candidates: 2014 – an update from the Pulmonary Transplantation Council of the International Society for Heart and Lung Transplantation.. J Heart Lung Transplant.

[7] Kerem E, Reisman J, Corey M, Canny GJ, Levison H (1992). Prediction of mortality in patients with cystic fibrosis.. N Engl J Med.

[8] Mayer-Hamblett N, Rosenfeld M, Emerson J, Goss CH, Aitken ML (2002). Developing cystic fibrosis lung transplant referral criteria using predictors of 2-year mortality.. Am J Respir Crit Care Med.

[9] Raghu G, Rochwerg B, Zhang Y (2015). An official ATS/ERS/JRS/ALAT Clinical Practice Guideline: Treatment of idiopathic pulmonary fibrosis. An update of the 2011 Clinical Practice Guideline.. Am J Respir Crit Care Med.

[10] Benza RL, Miller DP, Gomberg-Maitland M (2010). Predicting survival in pulmonary arterial hypertension: Insights from the Registry to Evaluate Early and Long-Term Pulmonary Arterial Hypertension Disease Management (REVEAL).. Circulation.

